# The Road to Sorghum Domestication: Evidence From Nucleotide Diversity and Gene Expression Patterns

**DOI:** 10.3389/fpls.2021.666075

**Published:** 2021-08-30

**Authors:** Concetta Burgarella, Angélique Berger, Sylvain Glémin, Jacques David, Nancy Terrier, Monique Deu, David Pot

**Affiliations:** ^1^CIRAD, UMR AGAP Institut, Montpellier, France; ^2^AGAP Institut, Univ F-34398 Montpellier, CIRAD, INRAE, Institut Agro, Montpellier, France; ^3^CNRS, Univ. Rennes, ECOBIO – UMR 6553, Rennes, France; ^4^Department of Ecology and Evolution, Evolutionary Biology Centre, Uppsala University, Uppsala, Sweden

**Keywords:** domestication, bottleneck, selection, nucleotide diversity, gene expression, sorghum

## Abstract

Native African cereals (sorghum, millets) ensure food security to millions of low-income people from low fertility and drought-prone regions of Africa and Asia. In spite of their agronomic importance, the genetic bases of their phenotype and adaptations are still not well-understood. Here we focus on *Sorghum bicolor*, which is the fifth cereal worldwide for grain production and constitutes the staple food for around 500 million people. We leverage transcriptomic resources to address the adaptive consequences of the domestication process. Gene expression and nucleotide variability were analyzed in 11 domesticated and nine wild accessions. We documented a downregulation of expression and a reduction of diversity both in nucleotide polymorphism (30%) and gene expression levels (18%) in domesticated sorghum. These findings at the genome-wide level support the occurrence of a global reduction of diversity during the domestication process, although several genes also showed patterns consistent with the action of selection. Nine hundred and forty-nine genes were significantly differentially expressed between wild and domesticated gene pools. Their functional annotation points to metabolic pathways most likely contributing to the sorghum domestication syndrome, such as photosynthesis and auxin metabolism. Coexpression network analyzes revealed 21 clusters of genes sharing similar expression patterns. Four clusters (totaling 2,449 genes) were significantly enriched in differentially expressed genes between the wild and domesticated pools and two were also enriched in domestication and improvement genes previously identified in sorghum. These findings reinforce the evidence that the combined and intricated effects of the domestication and improvement processes do not only affect the behaviors of a few genes but led to a large rewiring of the transcriptome. Overall, these analyzes pave the way toward the identification of key domestication genes valuable for genetic resources characterization and breeding purposes.

## Introduction

Domestication is an evolutionary process in which species evolve dramatic functional and phenotypic changes under selection for characters that fit the agricultural environment and human necessities (such as taste, yield, cultivation, harvesting and storage practices). The resulting domesticated forms are different from unmanaged wild populations and generally unable to reproduce and survive in the wild. The suite of traits that have been modified in the domesticated forms are collectively referred to as “domestication syndrome” (Hammer, 1984, as cited in Allaby, 2014). In plants they typically include characters related to seed dispersal (e.g., reduction of dehiscence and dormancy), plant architecture (e.g., branching) and properties of the harvested part (such as size, shape, nutritional content of seed, fruit and tubers). Around 2,500 plants are estimated to have experienced domestication with grasses (family *Poaceae*) having contributed the largest number (379, according to Dirzo and Raven, 2003).

The extensive phenotypic changes induced in domesticated species are associated with genetic changes, which can be put in evidence by comparing domesticated and wild forms. According to domestication models, an important genome wide signature of the domestication process is a global reduction of nucleotide diversity experienced by the domesticated forms. Recent studies including the once based on archeogenetics have given evidence that this process of reduction is protracted in time and includes different steps of genomic adaptation and deterioration throughout the history of cultivation of the species. The domestication's term that is used in the following sections of this article refers to the dynamic effects that led to the divergence of the current cultivated forms compared to the wild ones. This general reduction of genetic diversity is expected to be stronger for annual than perennial species and in autogamous than allogamous crops (Gaut et al., 2015). Experimental evidence supports this hypothesis, as a reduction of genetic diversity has been found in major domesticated species when compared with their wild progenitors, e.g., maize (Hufford et al., 2013), rice (Caicedo et al., 2007; Huang et al., 2012; Nabholz et al., 2014), soybean (Lam et al., 2010) and tomato (Koenig et al., 2013; Sauvage et al., 2017).

Besides genome-wide effects, the specific genomic regions involved in the phenotypic traits that distinguish domesticated from wild forms are expected to show stronger molecular genetic signatures than the rest of the genome. Although the genetic mechanisms under many complex domestication traits are still unknown, several major domestication genes have been identified along with their causative variation, which includes single nucleotide mutations, copy number polymorphism or indels changes (Kantar et al., 2017; Purugganan, 2019). These genes include both coding and regulatory regions, indicating that changes in expression levels may be as important as protein modifications (Meyer and Purugganan, 2013; Olsen and Wendel, 2013a,b). Supporting this hypothesis, recent surveys of gene expression patterns have put in evidence significant differences between the domesticated and the wild pools (e.g., Bellucci et al., 2014; Page et al., 2019). For example, in maize and tomato the rewiring of gene expression levels has been documented for some functional networks (Swanson-Wagner et al., 2012; Sauvage et al., 2017) up to shutdown complete pathways (Itkin et al., 2013), suggesting that the expression changes induced by domestication may have targeted metabolic pathways more than major effect genes (Sauvage et al., 2017). Domestication has also involved loss of connectivity within co-expression networks for some genes (Swanson-Wagner et al., 2012).

We focus here on sorghum (*Sorghum bicolor* [L.] Moench), the world's 5^th^ cereal for grain production. Being a staple food for more than 500 million people, sorghum plays a key role in food security in Africa and Asia. Aside from providing food for human consumption, sorghum is widely cultivated in all continents as a source for feed, fiber and energy (Paterson et al., 2009). In the current context of climate change and resource depletion, sorghum is also a cereal with great potential for future agriculture, because it outperforms other crops under low-input and stressful conditions (Hasan et al., 2017).

Sorghum was first domesticated in northeast-central Africa, likely around 5,000–6,000 years ago (Winchell et al., 2017, 2018; Fuller and Stevens, 2018). The domesticated phenotype is distinguishable from the wild forms in many morphological, physiological and phenological traits, including non-shattering grains, higher seed size, different panicle and plant architecture. Some genes and gene families have been identified as likely targets of the domestication and/or the improvement processes. Notably, the loss of seed shattering has been related to mutations in the *shattering1* (*sh1*) locus, which has likely undergone parallel selection in sorghum, rice and maize (Lin et al., 2012). Selection scans and phenotype-genotype association studies have identified other candidate genes. These include known cereal domestication genes Tb1 (*teosinte branched1*) and ba1 (*barren stalk1*), involved in plant architecture (Mace et al., 2013; Lai et al., 2018), as well as genes associated with grain features (e.g., Psy1, *phytoene synthase1*), photoperiod sensitivity (early maturity Ma genes) and plant height (Dw genes) (Fernandez et al., 2008; Mace and Jordan, 2010; Mace et al., 2013). Genes within key biosynthesis pathways have also been highlighted, tagging in particular the metabolisms of starch (ss1 and sbe3 genes, Campbell et al., 2016), grain size and weight (Tao et al., 2017, 2018).

In spite of its importance, there is still a limited knowledge about the genetic determinants of sorghum traits and adaptive potential. Comparing the domesticated form with its wild progenitor is particularly informative to understand the changes that accompanied the transition from the wild to the crop. By taking the wild pool as reference, we can quantify the genetic changes we observe in the domesticate both at the genome-wide scale and at the individual gene scale. Despite the simplicity of this approach, just a handful of studies have included wild accessions in analyzes of sorghum crop diversity and evolution in a comparative manner. These works have analyzed variation at allozyme markers (Aldrich et al., 1992), RFLP loci in the nuclear and chloroplast genomes (Aldrich and Doebley, 1992), nuclear microsatellite markers (Casa et al., 2005; Billot et al., 2013) and nucleotide sequence (Hamblin et al., 2005; Mace et al., 2013). They have all reported a global loss of genetic diversity and for some of them evidence of directional selection in the genome of landraces and improved germplasm.

In this work, we address the genetic bases of the transition from wild to domesticate in sorghum by analysing the transcriptome expressed in wild and domesticated accessions. Compared to nucleotide diversity screening, transcriptome analysis provides two-fold information about changes in the nucleotide sequence of coding genes and changes in their levels of gene expression. Here we perform complementary analyses to address these changes at the species level, with three main objectives: (i) to quantify the effect of domestication on nucleotide polymorphism, by looking at gene diversity and differentiation patterns; (ii) to unveil the effect of domestication on patterns of gene expression and document changes in single genes and gene networks; (iii) to assess the congruence and divergence of polymorphism and expression patterns to identify genes and pathways under selection and responsible for the evolution of the domesticated phenotype.

## Materials and Methods

### Plant Material

#### Brief Report on Taxonomy and Domestication

Numerous studies were conducted in the 1970's both to simplify the taxonomy of the S*orghum bicolor* species and to trace the origins and history of cultivated sorghums. Numerical taxonomy, distribution maps based on field observations and samples from herbaria of both cultivated and wild sorghum were used by the Crop Evolution Laboratory (University Illinois) to reach this goal (de Wet and Huckabay, 1967; de Wet and Harlan, 1971; Harlan and de Wet, 1972; de Wet et al., 1976). These authors grouped wild, weedy and cultivated sorghum in a single species, and described five basic races and 10 intermediate ones within the cultivated pool (*Sorghum bicolor* subsp. *bicolo*r) and four races within their progenitor wild pool *(Sorghum bicolor* subsp. *arundinaceum*, now renamed subsp. *verticilliflorum;* Doggett, 1988). The four wild races (aethiopicum, arundinaceum, verticilliflorum and virgatum), which differed primarily by their morphology and their ecological distribution (de Wet and Harlan, 1971) cross readily among them and with all cultivated races where they are sympatric. According to de Wet (1978), these wild races, better qualified as ecotypes with some overlap in their distribution, do not deserve formal taxonomic status. These four wild races were finally not validated in the recently revised nomenclature (Wiersema and Dahlberg, 2007).

Sorghum was first domesticated in northeast-central Africa from a complex of aethiopicum and/or verticilliflorum that likely gave an early bicolor type, which spread first into west Africa and then southern-east Africa and south Africa (de Wet et al., 1976; Harlan and Stemler, 1976; Fuller and Stevens, 2018). The bicolor type, through selection for local adaptation and probably introgression with local wild forms, in some regions gave rise to the four other races of cultivated sorghums (guinea in west Africa would be the earliest specialized types, while caudatum and kafir would be later domesticates). Finally, recent genetic studies suggested a possible separate domestication of guinea margaritiferum in west Africa (Deu et al., 1995; Sagnard et al., 2011; Mace et al., 2013). All these studies showed a complex pattern of sorghum taxonomy and domestication history and led us to not rule out any potential wild progenitor from our study.

#### Sampling Strategy

A data-set of 20 sorghum accessions were examined in this study. These accessions were selected to represent the diversity of the domesticated (*n* = 11) and the wild relative (*n* = 9) gene pools. The selection of the cultivated and wild accessions took into consideration their geographical origin and their racial information (although the relevance of the attributed races in the wild compartment remains debated as previously discussed). At the initial step of our selection, we took care to represent the wild accessions from northeast-central Africa, the supposed cradle of sorghum domestication, as well as other areas where introgression between wild and cultivated forms could occur. In addition, a verification of the grain morphological traits was also performed in order to avoid seedlot misclassification issues. Finally, a panel of 319 genotypes that included 207 cultivated genotypes from the CIRAD core collection (Deu et al., 2006) and 112 wild ones from *ex-situ* collections were genotyped by GBS (167 505 SNP after quality verification) and this information (M. Deu, personal communication) was used to refine the final selection. Grain morphological analysis combined with GBS results confirmed the passport information for 19 out of the 20 considered accessions (i.e., their membership to the wild and cultivated pools and their clustering with accessions from the same geographical origin and race for the cultivated accessions). For IS14719, which corresponds to a wild accession according to the passeport information (*S. b*. subsp. *verticilliflorum* race verticilliflorum from Ethiopia), morphological and genotyping analyses revealed a clear membership to the cultivated pool. Based on this assessment, this accession was considered as belonging to the cultivated pool in our analyses. Details on the accessions are provided in Supplementary Table 1, as well as the correspondence of accession names with published works analysing accessions from this data-set (Clément et al., 2017; Ranwez et al., 2017). All the genotypes were grown in the greenhouse until the mature grain stage. Tissue samples were harvested from three organs: mature leaves (fourth rank below the flag leaf), inflorescence (at the anthesis stage) and maturing grains (in average 25 days after anthesis).

### RNA Preparation and Sequencing

Each tissue was considered independently for RNA extraction, and RNA were then pooled for each accession prior to sequencing. RNA extraction, Illumina library preparation and sequencing conditions are detailed in Sarah et al. (2017). A mixture of 65% RNA from the inflorescence, 15% from leaves and 20% from grains for each accession was sequenced using Illumina mRNA-seq, paired-end protocol on a HiSeq2000 sequencer (one run for each genetic pool). The paired-end reads, in the illumina FASTQ format, were cleaned using cutAdapt (Martin, 2011) to trim read ends of poor quality (q score below 20) and to keep only those with an average quality above 30 and a minimum length of 35 base pairs (Sarah et al., 2017). Those data are freely available on the NCBI Sequence Read Archive database (SRA codes listed in Supplementary Table 1).

### Polymorphism Analysis

Reads were mapped on sorghum genome version 1.4 with the BWA software (Li and Durbin, 2009), allowing at most three mismatches between a given read and the reference by Sarah et al. (2017). To perform genotype calling, we followed the procedure of Nabholz et al. (2014). We excluded reads with more than two insertions/deletions (indels) or with indels larger than 5 bp or mapping on different transcripts. ORFextractor (available at http://fr.softwaresea.com/download-ORF-Extractor-10449769.htm) was used to extract coding sequences for further processing. The read2snp software (Gayral et al., 2013) was used to call genotypes and filter out paralogs. Following the approach of Nabholz et al. (2014) for non-outcrossing species, we did a first run setting the fixation index *F* = 0, then estimated *F* from the data and rerun the program once with the new estimated value. We note that the fixation index calculated here accounts for the mating system and potential substructure in each sample. For each individual and each position, we only kept genotypes with a minimum coverage of 10x and with posterior probability higher than 0.95. Otherwise, data was considered missing. The contigs obtained with reads2snp were further filtered for the downstream analysis.

The structure of genetic diversity revealed through the genotype calling steps was first explored using principal component analysis (PCA) with the function pca in package LEA v. 3.8 (Frichot and François, 2015). For this, we filtered the 24,474 contigs common to the wild and domesticated pools by discarding positions called in <5 individuals for each genetic pool, ending up with 11,699 contigs and 54,594 polymorphic loci. The same dataset was analyzed with popGenome (Pfeifer et al., 2014) to calculate population genomic statistics of diversity and differentiation: nucleotide diversity *π* and Tajima's *D* per pool, *F*_ST_ between pools. The fixation index *F* and polymorphism at synonymous (*π*_S_) and nonsynonymous (*π*_N_) sites were calculated with dNdSpiNpiS (available at https://kimura.univ-montp2.fr/PopPhyl/. In dNdSpiNpiS analysis, we started from 26,552 and 26,274 contigs in domesticated and wild pools respectively, and discarded positions called in < 5 individuals for each genetic pool, ending up with 13,231 and 11,397 contigs (Table 1).

**Table 1:** Characteristics of the data sets, coding sequence polymorphism and differentiation in wild and domesticated accessions of *Sorghum bicolor*.

	**Wild**	**Domesticate**
No. of accessions	9	11
No. of contigs retained by reads2snp	26,274	26,552
No. of contigs analyzed with dNdSpiNpiS	11,397	13,231
Mean length of retained contigs^a^ [95% quantiles]	1,610 [326; 3882]	1,596 [312; 3830]
No. of polymorphic sites^a^	46,147	41,179
*F* [95%CI]^a^	0.42 [0.408; 0.424]	0.99 [0.987; 0.989]
mean *π*^a^_S_ [95%CI]	4.1 [3.97; 4.17]	3.3^***^ [3.15; 3.35]
mean *π*^a^_S_ [95%CI]	0.7 [0.64; 0.67]	0.5^***^ [0.48; 0.51]
mean *π*_N_/*π*^a^_S_ [95%CI]	0.16 [0.156; 0.166]	0.15 [0.147; 0.158]
No. of contigs analyzed with PopGenome	11,699	
Length of retained contigs^b^ Mean [95% quantiles]	743 [0; 2600]	
No. of polymorphic sites^b^	32,712	24,453
Tajima's D^b^ Median [95% quantiles]	−0.4 [−1.6; 1.6]	−0.39^***^ [−1.8; 1.8]
mean *π* [95%CI]^b^	1.6 [0; 6.7]	1.1^***^ [0; 5.4]
Mean *π*_CROP_/*π*_WILD_ [95% quantiles]	0.65 [0; Inf]	
Mean F_ST_ [95% quantiles]^b^	0.07 [0; 0.42]	

*F: fixation index (Weir and Cockerham, 1984)*.

*π: nucleotide diversity (x 10^−3^). All polymorphic positions (π), synonymous sites (π_S_), non-synonymous sites (π_N_)*.

*F_ST_: pairwise differentiation (Hudson et al., 1992)*.

a *calculated with dNdSpiNpiS*.

b *calculated with popGenome*.

*** *Kolmogorov-Smirnov test for difference between domesticated and wild sorghum distributions, two-side p-value < 2.2e-16*.

### Differential Expression Analysis

Transcript expression levels were estimated with the new-Tuxedo pipeline (Pertea et al., 2016). Firstly, for each accession, RNA-seq reads were mapped on the sorghum genome assembly Sbicolor_313_v3.1 using Hisat2 (Kim et al., 2015). Genes and transcripts were assembled and quantified with stringtie (Pertea et al., 2015), using the reference annotation file to guide the assembly process. The output includes expressed reference transcripts as well as any novel transcripts that were assembled. Gffcompare (https://github.com/gpertea/gffcompare) was used to compare transcripts with the reference annotation (gene and transcript predictions) and to identify new genes/transcripts. Assembly and enriched annotation files were used to estimate abundance with stringtie (script provided as Supplementary Material Presentation 1).

Since we were interested in describing the transition from wild to domesticate at the species scale, we tested the differential expression of cultivated vs. wild considering the different individual genotypes within pools as replicates. The identification of differentially expressed genes between the two pools was done with edgeR (Robinson et al., 2010) under the R environment (R Core Team, 2018). Only genes assayed in at least five accessions and with at least one CPM (count per million) in each accession were included in subsequent analyses, considering that the expression level could not be reliably estimated for genes that did not meet these criteria. Since the minimum library size was 12 M, one CPM corresponded to 12 reads. A total of 24,646 genes passed these filters. To account for gene-specific expression biases at the individual level, a normalization step (trimmed mean of M-values (TMM) between each pair of samples) was performed (Robinson and Oshlack, 2010). The structure of the sample based on the gene expression levels was explored with a PCA on the normalized counts with the function dudi.pca of the R package ade4 (Dray and Dufour, 2007). Diversity in expression within each of domesticated and wild groups was quantified with the coefficient of variation (CV) in read counts. Domesticate-wild differences in CV were assessed in different gene categories (candidate and non-candidate genes under selection), to assess if the CV loss observed in domesticated sorghum was due to selection in the domesticated pool or drift associated to the domestication/improvement process, following the approach of Bellucci et al. (2014). Candidate genes were identified by extreme values of the polymorphism and differentiation statistics, non-candidates were all the other genes (Supplementary Table 2). The significance of the difference was assessed with 1,000 random resamplings.

To identify differentially expressed (DE) genes, gene-wise exact tests for differences in the means between the two groups (wild and domesticated sorghum) were performed. A False Discovery Rate approach was applied to correct for multiple tests. Thresholds of 0.05 and 0.01 FDR were considered to declare DE genes. At 0.05 FDR, we declared a number of DE genes two-fold higher than at 0.01 FDR, but the ratio of up- and downregulated genes was qualitatively similar, as well as the main categories of Gene Ontology annotation (see below). We therefore only report the results for the stringent FDR of 0.01.

### Polymorphism/Expression Comparisons

We compared patterns of gene expression and polymorphism to identify genetic signatures potentially linked to selection and the process of domestication. Since expression level can affect the intensity of selection (Drummond et al., 2005; Park et al., 2013; Nabholz et al., 2014), we looked at the correlation between polymorphism (synonymous, *π*_S_, non-synonymous, *π*_N_ and the ratio *π*_N_/*π*_S_) and expression in each gene pool, and tested for species difference by performing an analysis of variance including pools, expression level, and their interaction. Gene expression was estimated as the mean of normalized read counts across accessions within each gene pool. For these analyses, polymorphism values and expression level were log transformed.

We also compared the levels of diversity and differentiation between DE and non-DE genes, to detect signatures exclusive of DE genes and consistent with directional selection in the wild or the domesticated pool. In particular, we tested if DE genes were enriched for lower polymorphism values, or extreme values of Tajima's D or *F*_*ST*_. Domestication could have driven selection only on expression, with no changes in the nucleotide sequence. Thus, we also tested if expression divergence of DE genes was stronger than expected given their genetic divergence by testing if DE genes were enriched for *F*_ST_ = 0.

### Enrichment of Different Functional Categories

We examined the functional characterization of DE genes and assessed if particular gene categories or functions were overrepresented in DE vs. non-DE genes, which could be the result of selection. The categories assessed included the Gene Ontology (GO) categories, Sorghum transcription factors (retrieved from Plant Transcription Factor Database http://planttfdb.cbi.pku.edu.cn/index.php?sp=Sbi), stably expressed genes in sorghum (Shakoor et al., 2014), different metabolic pathways (Rhodes et al., 2014, 2017; Shakoor et al., 2014), domestication and improvement genes from published studies (Mace et al., 2013; Lai et al., 2018). Enrichment in the different functional categories was tested with Fisher's exact test on 2x2 contingency tables. GO annotation per gene was obtained from Sobic 3.1 genome annotation. The correspondence among gene models for the different versions of the Sorghum genome were retrieved with the conversion tool of the Grass Genome Hub (https://grass-genome-hub.southgreen.fr/pseudomolecules-converter). Functional annotation of novel assembled genes was done using Blast2GO (Götz et al., 2008). GO enrichment analysis was performed with topGO (Alexa et al., 2006) using the “classic” algorithm and Weighted Fisher's test to generate *p*-values. Map plots of non-redundant categories were done with REVIGO (Supek et al., 2011).

### Gene Expression Networks

Gene co-expression networks were built using the WGCNA R package (Langfelder and Horvath, 2008, 2012) using the normalized (TMM) and filtered expression data set (as previously described). Networks were built using the “signed” networkType parameter, enabling to capture the direction of the expression variation and grouping genes with the same direction variation in gene expression. This parameter is advised to identify biologically meaningful modules (van Dam et al., 2018). According to the mean connectivity and the scale free topology index curves obtained, a power of 20 was used for this analysis. A mergeheight parameter of 0.25 and a minimum module size of 30 were also considered. The modules obtained from this analysis were then tested for their enrichment in genes presenting differential expression patterns between the wild and domesticated pools.

## Results

### Nucleotide Diversity Patterns

For the analysis of polymorphism patterns the whole transcriptome of the wild and domesticated pools were analyzed separately (Table 1; Supplementary Table 2). Although a higher number of genes were retrieved for domesticated sorghum, a higher total number of polymorphic sites was called in the wild pool, suggestive of higher diversity in the wild populations (Table 1). Domesticated accessions showed a higher fixation index than wild sorghum (*F* = 0.99 and *F* = 0.42, respectively), in agreement with a higher rate of self-fertilization generally observed in the crop (e.g., Djè et al., 2004; Muraya et al., 2011).

**Figure 1:**
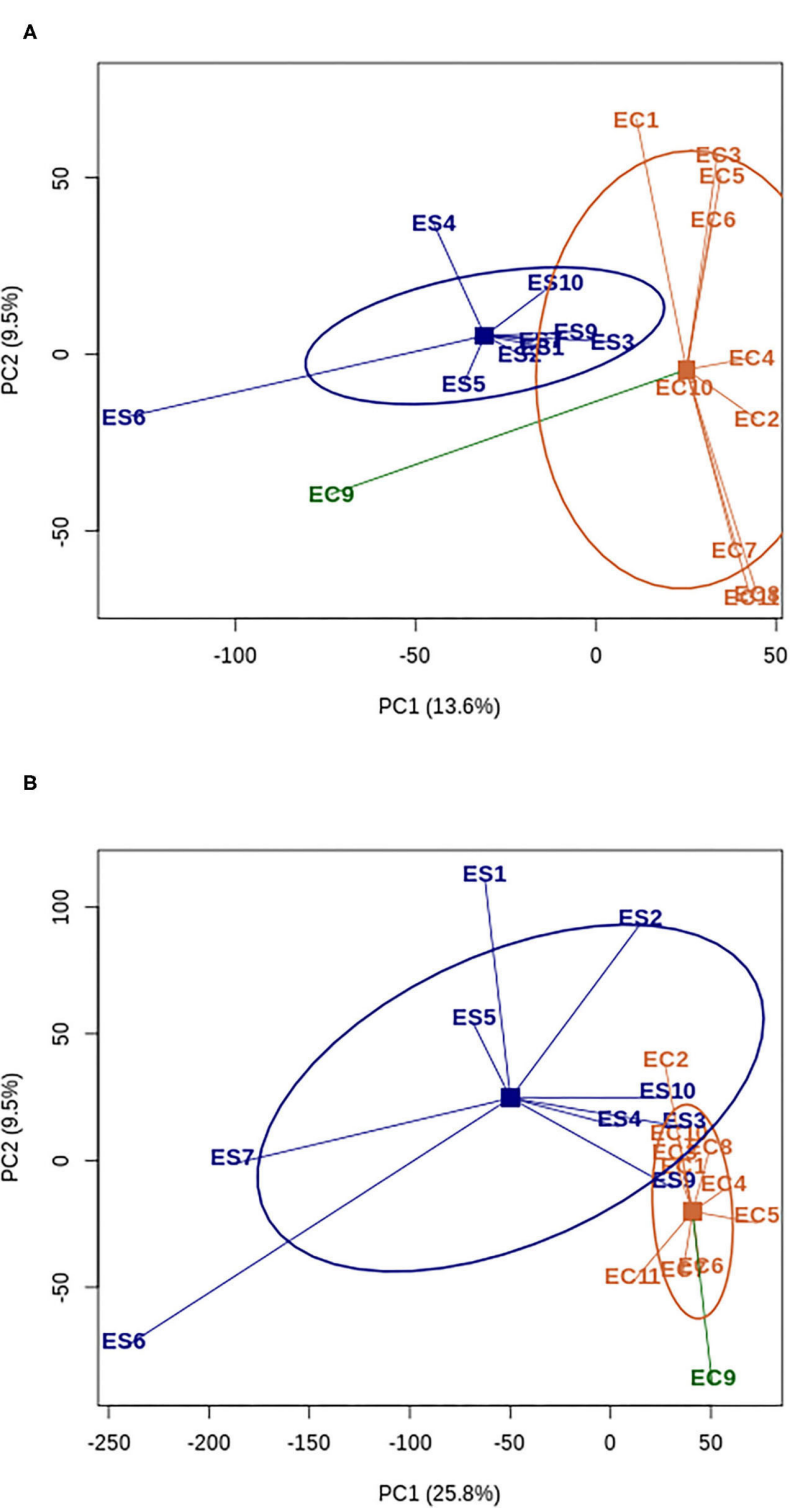
Principal Component analysis of polymorphism **(A)** and expression data **(B)** for wild (blue) and domesticated (orange) accessions of *Sorghum bicolor*. The accession EC9 corresponding to domesticated race guinea margaritiferum is represented with a different color (dark green) since its diversity patterns stand out from the domesticated pool. ES: wild; EC: domesticate. Squares represent the centroids of each group of accessions and ellipses represent the 60% 2D confidence interval.

The principal component analysis retrieved two genetic groups corresponding well to the wild and the domesticated pools (Figure 1). The only exception was the domesticated accession EC9 (SSM1057) that clustered with the wild samples. This accession belongs to the race guinea margaritiferum, which is cultivated but genetically close to the wild pool (Sagnard et al., 2011; Billot et al., 2013; Mace et al., 2013).

Domesticated sorghum harbored 20–30% less nucleotide diversity than the wild counterpart, in terms of number of polymorphic sites and nucleotide diversity (Kolmogorov-Smirnov test *p* < 2.2. e-16; Table 1). Non-synonymous polymorphism (*π*_N_) was six times lower than synonymous polymorphism (*π*_S_) in both domesticated and wild pools, in agreement with previous estimates for domesticated sorghum from Hamblin et al. (2006). We did not find evidence of accumulation of slightly deleterious mutations in domesticated sorghum at the genome scale, since the ratio of non-synonymous to synonymous polymorphism (*π*_N_/*π*_S_) across genes was similar in the wild and domesticated pools (mean values 0.16 and 0.15, respectively, Table 1).

We also estimated Tajima's *D*, which measures the departure of nucleotide diversity from neutrality due to selective or demographic processes. Domesticated and wild sorghum showed comparable close-to-zero mean Tajima's D values across genes (Table 1). Similar values were found genome-wide (Mace et al., 2013). The domesticated pool showed higher variance of Tajima's D gene estimates (Table 1). This can point to a higher number of genes departing from neutral equilibrium, although a higher stochastic variance associated with lower polymorphism cannot be ruled out.

We calculated the ratio *π*_crop_/*π*_wild_ on 7,853 genes. We considered the mean value *π*_crop_/*π*_wild_ = 0.65 (Table 1) to correspond to the global loss of genetic diversity due to the domestication bottleneck. Taking this value as reference, single gene *π*_crop_/*π*_wild_ ratio significantly different from the reduction of diversity expected during the domestication process may point to genes whose diversity has changed because of selection. Since strong positive selection is expected to reduce diversity in the genomic region linked to the causal allele, *π*_crop_/*π*_wild_ < 0.65 would characterize genes under positive selection in the domesticate. In particular, strong domestication candidates are genes that are monomorphic in the domesticate and polymorphic in the wild pool. Conversely, *π*_crop_/*π*_wild_ > 0.65 may indicate a diversity increase following domestication, either due to other selective dynamics (e.g., balancing selection or diversifying selection across varieties) or to a relaxation of selection in the domesticated pool compared to the wild one. We found that 1,734 genes (22%) showed *π*_crop_/*π*_wild_ = 0 and 2,500 (32%) had *π*_crop_/*π*_wild_ > 1.

Finally, we estimated the genetic differentiation between domesticated and wild sorghum by calculating *F*_*ST*_ on a gene by gene basis. Overall differentiation was quite low (mean *F*_*ST*_ = 0.07, Table 1), and did not change when genes with <5 polymorphic sites were excluded. Our estimate is lower than previous estimates (*F*_*ST*_ = 0.13 on SSRs, Casa et al., 2005). Two reasons can be proposed here to explain this discrepancy. Firstly, the coding regions analyzed in this study could have experienced a lower neutral drift than the microsatellite markers used by Casa et al. (2005). Secondly, it can emerge from the different compositions of the panels used. Indeed, we included a guinea margaritiferum accession in our domesticated pool whereas such type of accession was, to the best of our knowledge, not used in the analysis of Casa et al. (2005).

### Expression Diversity Patterns

Reads counts were found for 38,885 genes, with 95% of them with read counts in either the domesticated or the wild pool. The analysis of gene expression levels was done on 24,646 genes meeting the criterion of at least one read count per million in at least five samples (Supplementary Table 2). Overall, our sampling strategy (three different tissues) allowed accessing more than 70% of the sorghum transcriptome. Similarly to the nucleotide diversity information, to compare the diversity in expression between domesticated and wild sorghum, we performed a principal component analysis and calculated the coefficient of variation (CV) on normalized read counts. PC1 separated the domesticated and wild pools, explaining 25.8% of the read counts diversity (Figure 1). The domesticated pool harbored 84% of the wild pool expression variability (mean CV_crop_ = 0.63 [95% quantiles 0.13; 1.98], mean CV_wild_ = 0.75 [95% quantiles 0.16; 2.04]), which suggests that domestication in sorghum involved also a loss of expression diversity, besides the loss of polymorphism reported above. We found that the CV reduction is higher (at least 25%) for candidate genes harboring *F*_ST_ excess and diversity reduction (potential signatures of selection) at the nucleotide level irrespective of the statistic used to identify them. This signal is stronger for genes with extremely high domesticate-wild differentiation. The CV loss was 34%, 32% and 29% in the 99%, 95% and 90% percentile of *F*_ST_ values respectively (Supplementary Figure 1). To exclude that this stronger CV reduction was simply due to a lower nucleotide diversity in *F*_ST_ outlier genes by comparison with non-outlier ones, we calculated the difference CV_wild_-CV_crop_ for 1,000 random samples of non-outlier genes with equal or lower diversity level (*π*) than outlier genes. Wild to domesticate CV reduction in *F*_ST_ outliers was significantly higher than the CV reduction obtained with the random samples for the three *F*_ST_ percentiles (*p*-value < 0.001 for 95% and 90% and *p*-value < 0.01 for 99%; Supplementary Figure 2).

**Figure 2:**
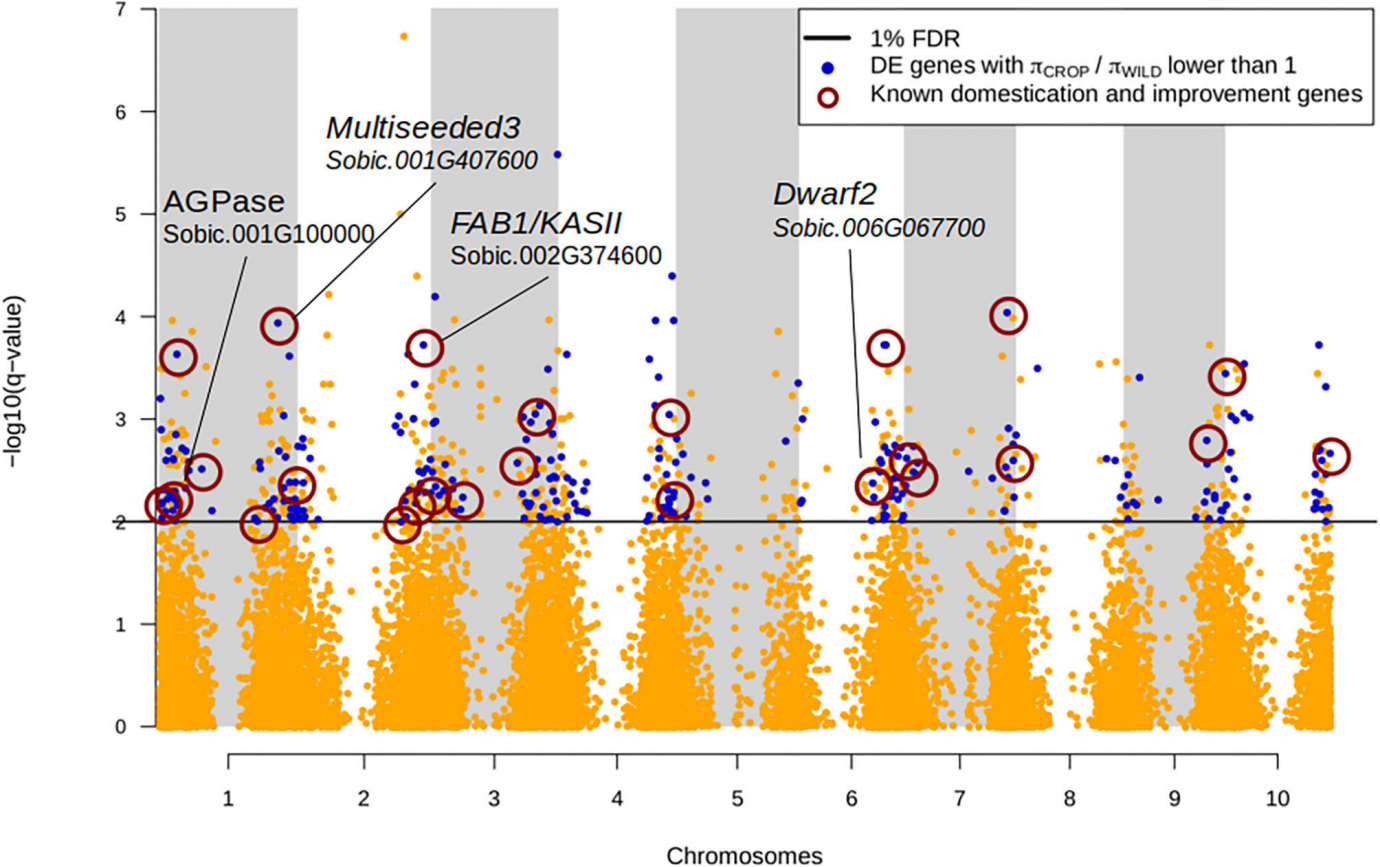
Manhattan plot of FDR *q*-values associated with the differential expression analysis between wild and domesticated sorgho. Genes were declared significantly differentially expressed at 1% FDR (black line). Genes with lower nucleotide diversity in the domesticated pool (π_CROP_/*π*_WILD_) are represented in blue. Among them, 70 are known candidate domestication/improvement genes (dark red circles, Supplementary Table 3 and ref. therein). The position of four genes affecting the domesticated phenotype is also represented.

We tested for significantly different expression levels between the wild and domesticated pools, to identify genes whose change in expression may have been driven by the process of domestication. We found 949 differentially expressed (DE) genes at a FDR of 1% (Figure 2; Supplementary Table 4; Supplementary Figure 3). The difference in expression levels corresponded to |logFC| > 0.55 (logFC range: −9 to 14). Interestingly, among the DE genes, 82% were down-regulated in domesticated sorghum, while we did not observe any bias in the direction of expression change in the whole gene set (χ^2^ test, *p* < 2e-16). This result is qualitatively similar for a FDR of 5% (among 2,291 DE genes, 72% were down-regulated in domesticated sorghum; Supplementary Figure 4).

### Comparison of Expression and Nucleotide Diversity Patterns

We first compared expression and nucleotide patterns across all genes. We found that gene polymorphism (either estimated with *π*_S_, *π*_N_ or *π*_N_/*π*_S_) was negatively correlated with expression (Table 2). Negative correlation between expression level and *π*_N_ or *π*_N_/*π*_S_ is expected if higher expressed genes experience stronger selection against new deleterious mutations, whereas this pattern for *π*_S_ may suggest other processes in action (e.g., selection on code usage). For *π*_N_/*π*_S_, the correlation was less negative for the domesticated accessions, which may be the effect of weaker purifying selection and domestication load experienced in the domesticated pool. Indeed, the analysis of variance showed that the relationship between polymorphism and expression is significantly different between the two genetic pools for all *π* estimates (Supplementary Table 5); a difference potentially due to the process of domestication.

**Table 2:** Correlations of polymorphism estimates and expression levels.

		**Slope**	**adjR^**2**^**	***p*-value**
*π*_S_	Domesticate	−0.39	0.13	<2e-16
	Wild	−0.26	0.06	<2e-16
*π*_N_	Domesticate	−0.54	0.24	<2e-16
	Wild	−0.44	0.18	<2e-16
*π*_N_/*π*_S_	Domesticate	−0.14	0.014	<2e-12
	Wild	−0.19	0.03	<2e-16

*π: nucleotide diversity at synonymous sites (π_S_) and non-synonymous sites (π_N_)*.

Then we looked at specific features of DE vs. non-DE genes to identify signatures associated with differential expression. DE genes showed values of π_N_ and π_N_/π_S_ lower than non-DE genes in both domesticated and wild pools (Table 3), suggesting stronger purifying selection on DE genes, although the distributions were significantly different only for π_N_ in domesticated sorghum, (Kolmogorov-Smirnov test *p*-value = 0.008). DE genes were also characterized by significantly lower values of domesticate to wild π ratio than non-DE genes (*p*-value = 0.02 and 0.04 for π_N_ and π_S_, respectively). This difference was largely driven by genes that are monomorphic in domesticated sorghum, which may represent genomic regions targeted by human-driven directional selection. DE genes also showed higher Tajima's D values than non-DE genes, but the difference was significant only in the wild pool (*p*-value = 0.024). We did not find significant differences between DE and non-DE genes for π_S_ or crop-wild *F*_ST_ (Table 3). Enrichment tests were, in general, consistent with distribution comparisons. DE genes were significantly enriched of π_crop_/π_wild_ values lower than 0.1 (corresponding to the lowest 20th percentile; Fisher exact test *p*-value = 0.03) and *F*_ST_ ≥0.35 (top 5^th^ percentile; *p*-value = 0.0014), Figure 2. Furthermore, among DE genes, 187 showed *F*_ST_ = 0; these genes are good candidates of domestication-driven expression divergence involving only regulation changes without coding sequence divergence (Figure 2; Supplementary Figure 5).

**Table 3:** Comparison of polymorphism patterns between genes significantly differentially expressed (DE) at 1% FDR and non-differentially expressed genes (non-DE) between wild and domesticated *Sorghum bicolor*.

	**DE genes (** ***n*** **=** **949)**		**Non-DE genes (** ***n*** **=** **23697)**	
	**up-regulated in Domesticate**	**down-regulated in Domesticate**	**Wild**	**Domesticate**
No. Genes	176	773^c^	12,410	11,287
mean π^a^_S_ [95%CI]	4.67 [0.03; 20.5]	3.73 [0.03; 24.9]	4.09 [0.03; 22.7]	3.28 [0.03; 21.5]
mean π^a^_S_ [95%CI]	0.61 [0.03; 4.1]	0.48^**^ [0.03; 3.8]	0.65 [0.03; 4.5]	0.5^**^ [0.03; 3.4]
mean π_N_/π^a^_S_ [95%CI]	0.13 [0; 1.1]	0.13 [0; 1]	0.16 [0; 1.1]	0.15 [0; 0.97]
Tajima's D^b^ Median [95% quantiles]	−0.25^*^ [−1.5; 1.5]	−0.19 [−1.6; 1.7]	−0.31^*^ [−1.5; 1.4]	−0.26 [−1.7; 1.6]
Median π_N−CROP_/π_N−WILD_	0.799^*^		0.801^*^	
Median π_S−CROP_/π_S−WILD_	0.767^*^		0.769^*^	
Mean ${\text{F}}_{\text{ST}}^{\text{b}}$ [95% quantiles]	0.09 [0; 0.4]		0.07 [0; 0.3]	

a *calculated on 469 genes with polymorphism estimates*.

b *calculated on 9,867 genes with polymorphism estimates*.

c *Among DE genes, the number of genes down-regulated in the domesticated pool is significantly higher than among non-DE genes (χ^2^ test p-value < 0.001)*.

* distribution significantly different in DE vs. non-DE genes (Kolmogorov-Smirnov test ^*^p-value < 0.05,

** *p-value < 0.01)*.

*π: nucleotide diversity (x 10^−3^). synonymous sites (π_S_), non-synonymous sites (π_N_)*.

### Biological Functions of Differentially Expressed Genes

Gene ontology (GO) analysis of DE genes revealed that terms related to photosynthesis were largely overrepresented among genes with reduced expression in the domesticated relative to the wild sorghum (*n* = 51 genes, Supplementary Table 6; Supplementary Figure 6). Different metabolic functions were instead enriched in DE genes upregulated in the domesticate, the more recurrent being related to cytoskeleton organization and intracellular membrane transport (Supplementary Table 7; Supplementary Figure 7). Genes upregulated in the domesticated pool were enriched also in GO terms associated with Auxin responsive factors (ARF, GO:0005086 and GO:0032012). Gene functional annotation for all genes analyzed in this study is provided in Supplementary Table 8.

We tested DE enrichment in Sorghum transcription factors (Plant Transcription Factor Database http://planttfdb.cbi.pku.edu.cn/index.php?sp=Sbi) and genes from the Sorghum expression Atlas (Shakoor et al., 2014). We found that DE genes were significantly depleted of transcription factors (*p*-value = 0.009) and of genes reported to be stably expressed across tissues (*p*-value = 3.462e-05) compared to non-DE genes.

We also compared our DE gene list with the candidate genes involved in trait domestication and improvement of sorghum according to previous studies. We found 59 genes harboring either a domestication or improvement signature as reported in Mace et al. (2013) and Lai et al. (2018) (Figure 2; Supplementary Table 3).

Several DE genes belonged to known metabolic pathways in sorghum. Among them there were two genes involved in the sucrose metabolism and 4 involved in the phenylpropanoyl monolignol pathway according to Shakoor et al. (2014), but these metabolisms were not more represented in DE than in non-DE genes. Other DE genes were previously known to be involved in the biochemical pathways associated with grain development and filling phases (Supplementary Table 3), such as starch synthesis (Sobic.001G100000, Campbell et al., 2016), polyphenols synthesis (four genes, Rhodes et al., 2014) and more generally grain composition (nine genes, Rhodes et al., 2017).

### Gene Co-expression Networks

Gene co-expression network analyses revealed 21 clusters of genes sharing similar expression patterns (Supplementary Table 9). Four of these clusters were enriched in differentially expressed genes between the wild and domesticated pools. Three were characterized by down-regulation in cultivated sorghum and a higher variability in wild sorghum (yellow: 2,051 genes, magenta: 173 genes and cyan: 92 genes) and one was characterized by up-regulation and higher variability in domesticated sorghum (greenyellow: 133 genes) (Supplementary Table 10). Among these four clusters, two were also enriched in domestication and improvement candidate genes previously identified through whole genome sequencing of 44 sorghum accessions including 7 wild and weedy genotypes (Mace et al., 2013) (Supplementary Tables 11, 12). More specifically, the yellow and greenyellow modules were enriched in domestication genes whereas the greenyellow module was also enriched in improvement genes. Interestingly, the greenyellow module that harbored an enrichment in improvement genes presented up-regulation in the domesticated pool compared to the wild pool. In addition, the black (271 genes) and the lightcyan (45 genes) modules harbored expression patterns revealing the specific properties of the guinea margaritiferum accession (SSM1057). In the black module SSM1057 harbored up-regulation in comparison to all the other accessions analyzed, whereas in the lightcyan module SSM1057 presented an expression pattern more closely related to the wild pool (although it is a domesticated sorghum).

## Discussion

In this study, we have performed a comparison of nucleotide and expression patterns between wild and domesticated forms of sorghum, to contribute to the understanding of the genetic circumstances of wild-to-domesticate transition in this major cereal. By targeting the full transcriptome, we focused on genes actually expressed, narrowing the analysis of interest to the part of diversity potentially most relevant.

### Down-Regulation and Diversity Loss During Sorghum Domestication

Our genome-wide screen of expressed genes showed a loss of diversity in the domesticated pool up to 30%, both in nucleotide and in expression diversity, supporting the general model of sorghum domestication from a reduced representation of the wild pool (e.g., Hamblin et al., 2006) and serial founder effects combined with different adaptation and deterioration steps during crop evolution (Wang et al., 2017; Allaby et al., 2019; Smith et al., 2019). Our estimate of nucleotide diversity loss was higher than previous estimates for domesticated sorghum based on nuclear microsatellite markers (e.g., 14%, Casa et al., 2005), but lower than the loss recorded in other main crops with selfing or mixed mating systems. For instance, it was estimated a 62% nucleotide diversity reduction in rice (Caicedo et al., 2007), 72% in common beans (Bitocchi et al., 2013) and 50% in soybean (Kim et al., 2021). Domesticated sorghum also showed lower expression variation than the wild accessions, a finding common to other crops such as maize (Lemmon et al., 2014) and common bean (Bellucci et al., 2014) but also animal domesticates (Liu et al., 2019). We demonstrated that the loss of expression diversity is not merely a consequence of the observed loss of coding nucleotide diversity. In fact, the genes with strongest signatures of selection showed greater loss of expression variation, which suggests that selection has driven strongest changes in particular genes, a finding common to other domesticate species (Bellucci et al., 2014; Liu et al., 2019).

In nature, changes of gene expression are responsible for a great part of phenotypic divergence not explained by amino acid divergence (Carroll, 2008). It is also likely that part of the phenotypic evolution associated with the domestication process has involved gene regulation without changes in the coding sequence. This appears to be the case in sorghum, where many DE genes do not show different nucleotide diversity patterns between the domesticated and the wild pools, suggesting that domestication left a footprint in expression but not in protein composition for these genes.

Our differential expression analysis indicated that domestication largely favored the down-regulation of expression of multiple genes in sorghum crop (82% of DE genes). In agreement with our findings in sorghum, common bean and eggplant landraces showed downregulation of expression when compared with wild relatives, although the analysis was limited to the leaf transcriptome in both studies (Bellucci et al., 2014; Page et al., 2019). In maize, Lemmon et al. (2014), analyzing three different tissues (leaf, ear, stem) found the opposite trend, with differentially expressed genes showing higher expression of the maize allele more often than the teosinte allele. In contrast, Sauvage et al. (2017) found a similar number of down- and up-regulated genes in domesticated tomatoes based on the analysis of vegetative and reproductive tissues (leaf, flower and fruits). The lack of consistent patterns across different crops suggest species-specific processes and mechanisms for the evolution of the domesticated phenotype.

### Biological Significance of Differentially Expressed Genes and Genes Harboring Putative Signature of Selection at the Nucleotide Level

In this study, we found 949 differentially expressed genes between the wild and domesticated pools taking advantage of leaves, flowers and maturing seeds of sorghum. Most of the genes were down-regulated in domesticated sorghum. Among them, genes with function associated with photosynthesis were disproportionately represented, suggesting that photosynthesis may be a key metabolic pathway contributing to the sorghum domestication syndrome. A number of studies have investigated the effect of domestication on photosynthesis metabolism, suggesting a shift in plant functional strategies over the course of domestication. For instance, higher net photosynthetic rate has been found in modern varieties of wheat compared to ancestral ones (Roucou et al., 2018) and in cultivated cassava compared with wild forms (Pujol et al., 2008), pointing to selection for increased photosynthetic efficiency during domestication and improvement. Genes significantly downregulated in domesticated sorghum suggested also that antioxidative responses have been weakened by domestication. Consistently with this scenario, it has been shown that ascorbate, an antioxidant widely associated with photosynthetic functions and stress tolerance, was reduced by domestication in some crop species, likely as a tradeoff of selection for higher fruit size and yield (Gest et al., 2013).

Modifications of the photosynthetic metabolism may be also a consequence of changes in the selective pressure experienced by the crop under some stresses. For instance, photosynthetic activity in leaves is overall reduced under drought stress (Osakabe et al., 2014). Some authors have suggested that the transition from wild habitats with unpredictable and scarcer water availability to agricultural environments with more regular and abundant water supplies led to a relaxation of selection for water use efficiency while favoring the increase of CO_2_ uptake and evaporative cooling (Milla et al., 2013 and references therein). It can be argued that domestication has led to a relaxation of selection for photosynthesis efficiency under drought stress, because of higher water availability in agricultural ecosystems than in the wild. This scenario would be consistent with positive selection for relatively higher photosynthetic capacity in cultivars adapted to water deficit environments, such as the case of upland rice compared with lowland rice (Zhang et al., 2016).

Among DE genes, a small number was upregulated in domesticated sorghum (176). These genes were enriched in Auxin responsive factors, which are very likely drivers of domestication-related phenotype. Genes involved in the response to auxin stimulus contribute to abiotic stress response in sorghum (Wang et al., 2010) and more generally is part of the auxin signaling machinery determining the development of branches and flowers in major crops such as maize (Galli et al., 2015). Auxin-responsive genes have been found under selection in sorghum, maize, rice and domesticated emmer (Mace et al., 2013; Meyer and Purugganan, 2013; Avni et al., 2017).

Altogether, our scan for selection signatures and differential expression identified several individual candidate genes particularly interesting for their potential role in the domestication and diversification of cultivated sorghum (Figure 2). Some of these genes are already known to contribute to important phenotypic traits in sorghum or have been previously identified as sorghum domestication genes. For instance, the gene Sobic.006G067700 (*dwarf2*) has been reported to control the internode length in domesticated sorghum and to have pleiotropic effects on panicle length, seed weight and leaf area (Hilley et al., 2017). This gene was upregulated in the domesticated pool (logFC = −0.95, *q*-value = 4.14E-03), consistently with its involvement in the evolution of the domesticated phenotype. We detected also *multiseeded3* gene (MSD3, Sobic.001G407600, Chr01:69162600.69165731), a plastidial ω-3 fatty acid desaturase involved in the regulation of grain number in sorghum (Dampanaboina et al., 2019) and identified as domestication gene (Supplementary Table 3; Mace et al., 2013; Lai et al., 2018). This gene was downregulated in the domesticated pool (logFC = 1.99, *q*-value = 0.11E-03) and shows a strongly reduced polymorphism in the domesticated pool (π_crop_/π_wild_ = 0.4). Another interesting gene is Sobic.004G272100, which encodes a phosphoribulokinase, an enzyme that catalyzes a key step in carbon fixation as part of the Calvin cycle (dark phase of photosynthesis). This gene has been found to be under selection in sorghum and maize (Lai et al., 2018). We found it was down-regulated in domesticated sorghum (logFC = 1.86; *q*-value = 0.0096), suggesting that selection has targeted the reduction in expression level for this gene. In addition to the genes previously detected in the literature with evidence of signature of selection, our studies also provided a large list of additional genes that contributed or have been impacted by the domestication and improvement process. To further explore the list of genes that potentially contributed to these two key steps of crop evolution we studied their co-expression patterns.

### Domestication and Improvement Steps Involve Major Rewirings of Gene Expression

Gene co-expression network analyses allowed the identification of four clusters of genes (totalizing 2,449 genes) significantly enriched in differentially expressed genes between wild and domesticated sorghum. Two of these clusters were also enriched in domestication and improvement genes previously identified in sorghum according to their nucleotide diversity patterns. These results reinforce the conclusion that can be drawn from the differential expression analysis indicating that domestication and improvement do not only affect the behavior of a few “domestication or improvement” genes but led to large rewirings of the transcriptome during the domestication event and the improvement process, as also underlined in tomato (Sauvage et al., 2017) and cotton (Gallagher et al., 2020). Whereas the “gene based” differential expression analyses can be limited by the sequencing depth achieved for each specific gene to detect significant expression differences, the co-expression gene network approach provides a complementary way to identify additional potentially relevant genes.

For RNA sequencing, we pooled together leaf, flowers and maturing seeds tissues in the same proportions for each genotype. The simultaneous analysis of different tissues gives access to a wider set of genes than each tissue alone, and thus allows a better coverage of the genome-wide impact of domestication at the transcriptome level. However, with this approach we may have lost some power to identify differential expression patterns in very specific cases, e.g., in genes that experienced opposite expression evolution in the different tissues. While the approach taken here doesn't reduce the significance and the biological relevance of the genes we were able to detect, further work exploring independently the expression patterns in the different tissues may enlarge the list of candidate genes involved in the domesticated phenotype.

In the evolutionary history of sorghum, the guinea margaritiferum emergence remains until now quite unclear. Recent works proposed that they can result from a second and more recent domestication in West Africa (Sagnard et al., 2011; Mace et al., 2013). Our results indicated that the guinea margaritiferum accession presented a high similarity with the wild pool at the nucleotide level as previously reported, e.g., by Mace et al. (2013) and Morris et al. (2013). However, at the expression level, it behaved essentially as a domesticated genotype. Assuming a relatively recent domestication of the guinea margaritiferum sub-race, such results would indicate that the domestication process induced a rapid rewiring of the gene co-expression network and a much slower response at the genome-wide nucleotide level. It is also interesting to note that among the two clusters of genes for which an atypical behavior of the guinea margaritiferum was observed in comparison to the other domesticated genotypes (the black and lightcyan modules), there is the gene Sobic.001G341700, which has already been shown to contribute to the grain size in sorghum (Tao et al., 2017, 2020). This gene is an ortholog of Os DEP1 which has been proved to impact grain yield in rice (Huang et al., 2009).

## Conclusion

This work highlighted the major impact of the transition from the wild to the cultivated pools not only on the nucleotide diversity but also on the transcriptome landscape. Indeed, it revealed a major rewiring of the gene co-expression networks between the wild and the crop pools. In addition to these global observations, these analyses also identified specific metabolic pathways (photosynthesis, auxin related mechanisms) that are likely to have contributed to the transition from the wild to the crop pools in sorghum. Finally, our results also contributed to a better understanding of the specific attributes of the guinea margaritiferum genotypes within the sorghum genetic diversity highlighting the specific role of the transcriptome regulation in the emergence of the “domesticated” ideotype.

Although the set of accessions analyzed here is relatively limited (9 wild and 11 cultivated accessions), it allowed us to document genetic changes associated with the domestication and improvement process in sorghum at the global level (i.e., at the species scale). Future studies with more extensive sampling of the wild and cultivated pools will allow refining the scenarios at more precise geographical and racial scales. If the recent advances of the archaeogenetics emerging field will improve our understanding of the transition from the wild to the domesticated pools at the nucleotide diversity level, generalization of their results will require access to a wider number of archaeological sites. It is also important to stress that combined efforts based on archeogenetics and the use of contemporary accessions from the two pools will be required to refine our global understanding (i.e. nucleotide and transcriptome diversity) of the impacts of the transition from wild to cultivated pools. Indeed, analysis of transcriptome evolution between wild and cultivated pools will only partially benefit of archaeogenetics analyses as only the diversity of the transcription factor and the regulating regions of the coding genes will be accessible, leaving the global image of the co-expression networks evolution out of reach.

## Data Availability Statement

Publicly available datasets were analyzed in this study. This data can be found here: NCBI Sequence Read Archive database: SAMN05277472 to SAMN05277481; SAMN06052464 to SAMN06052472 and SAMN07313361. Corresponding sample metadata are provided in Supplementary Table 1.

## Author Contributions

CB performed the analyses, interpreted the results, wrote the initial version of the manuscript, aggregated the suggestions of the co-authors and finalized the manuscript. AB managed the production of the tissues, took care of the RNA extraction and took part to the gene expression analyses. SG wrote and led the ARCAD project together with JD, contributed to the SNP calling section and contributed to the improvement of the manuscript. JD wrote and led the ARCAD project together with SG and contributed to the improvement of the manuscript. NT contributed to the interpretation of the differential expression results, contributed to the improvement of the manuscript. MD contributed to the interpretation of the nucleotide diversity results, and took part to the revision of the manuscript. DP contributed to the writing of the sorghum section of the ARCAD project, led the sorghum activities of the BFF project, selected the genotypes that have been used in the current study, took part in the analyses, contributed to the writing of the manuscript and co-led with CB the finalization of the manuscript. All authors contributed to the article and approved the submitted version.

## Conflict of Interest

The authors declare that the research was conducted in the absence of any commercial or financial relationships that could be construed as a potential conflict of interest.

## Publisher's Note

All claims expressed in this article are solely those of the authors and do not necessarily represent those of their affiliated organizations, or those of the publisher, the editors and the reviewers. Any product that may be evaluated in this article, or claim that may be made by its manufacturer, is not guaranteed or endorsed by the publisher.
