# Supplementary material for: The Road to Sorghum Domestication: Evidence From Nucleotide Diversity and Gene Expression Patterns
Source: Front Plant Sci. 2021 Aug 30;12:666075. doi: 10.3389/fpls.2021.666075 (PMC8435843; doi:10.3389/fpls.2021.666075)
Supplement: Supplementary file 1 [file Data_Sheet_1.zip › Suplementary_Figure_S1.pdf]

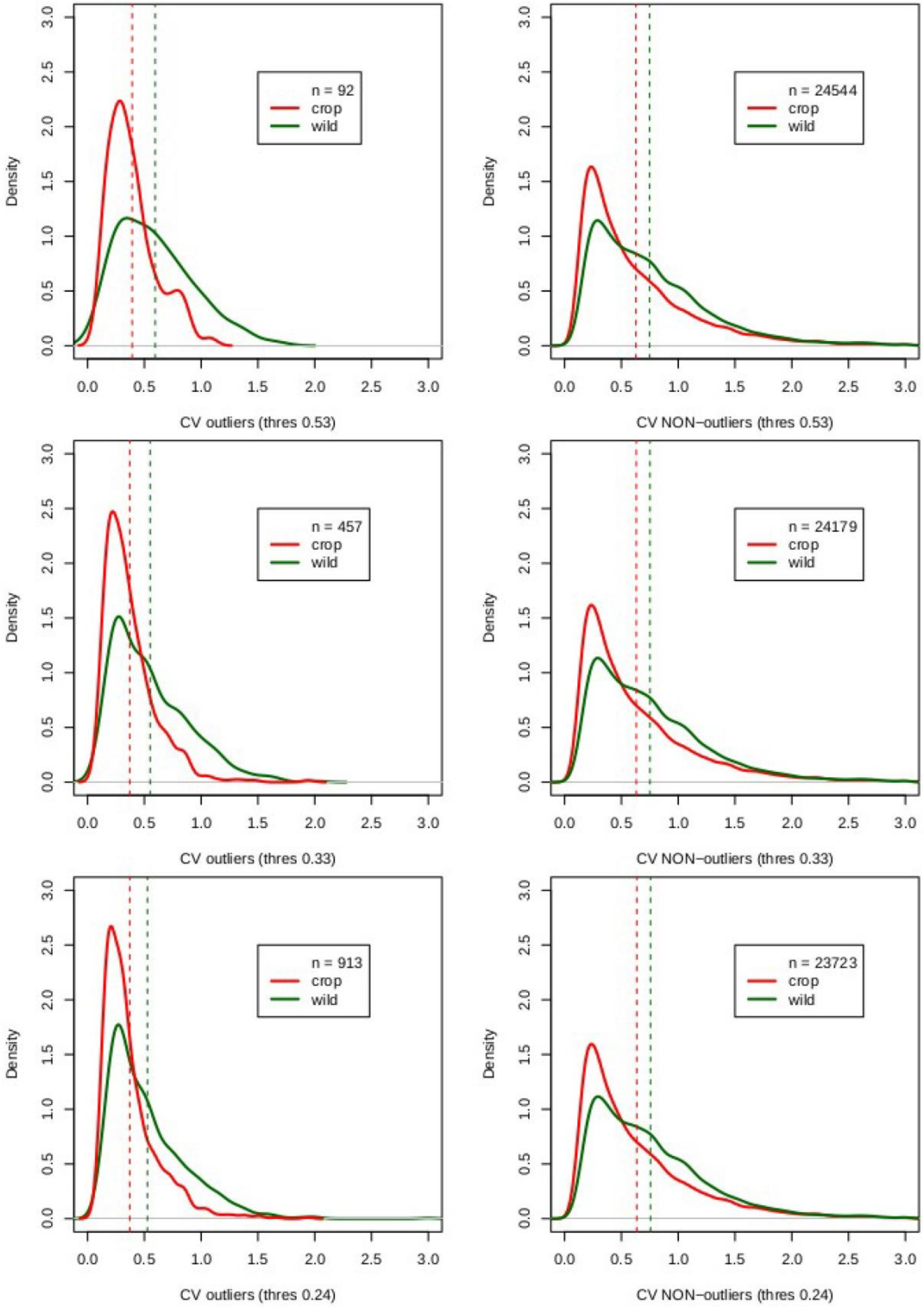

Figure S1. Density distribution of the coefficient of variation (CV) in expression for two gene categories. On the left, genes with extreme values of crop-wild differentiation (*F*ST outliers) identified at three percentile thresholds: 99%, 95%, 90% (from upper to bottom). On the right, all remnant genes (non-outliers). CV reduction was calculated as 1-(mean CVCROP / mean CVWILD).
