# Supplementary material for: The Road to Sorghum Domestication: Evidence From Nucleotide Diversity and Gene Expression Patterns
Source: Front Plant Sci. 2021 Aug 30;12:666075. doi: 10.3389/fpls.2021.666075 (PMC8435843; doi:10.3389/fpls.2021.666075)
Supplement: Supplementary file 1 [file Data_Sheet_1.zip › Suplementary_Figure_S2.pdf]

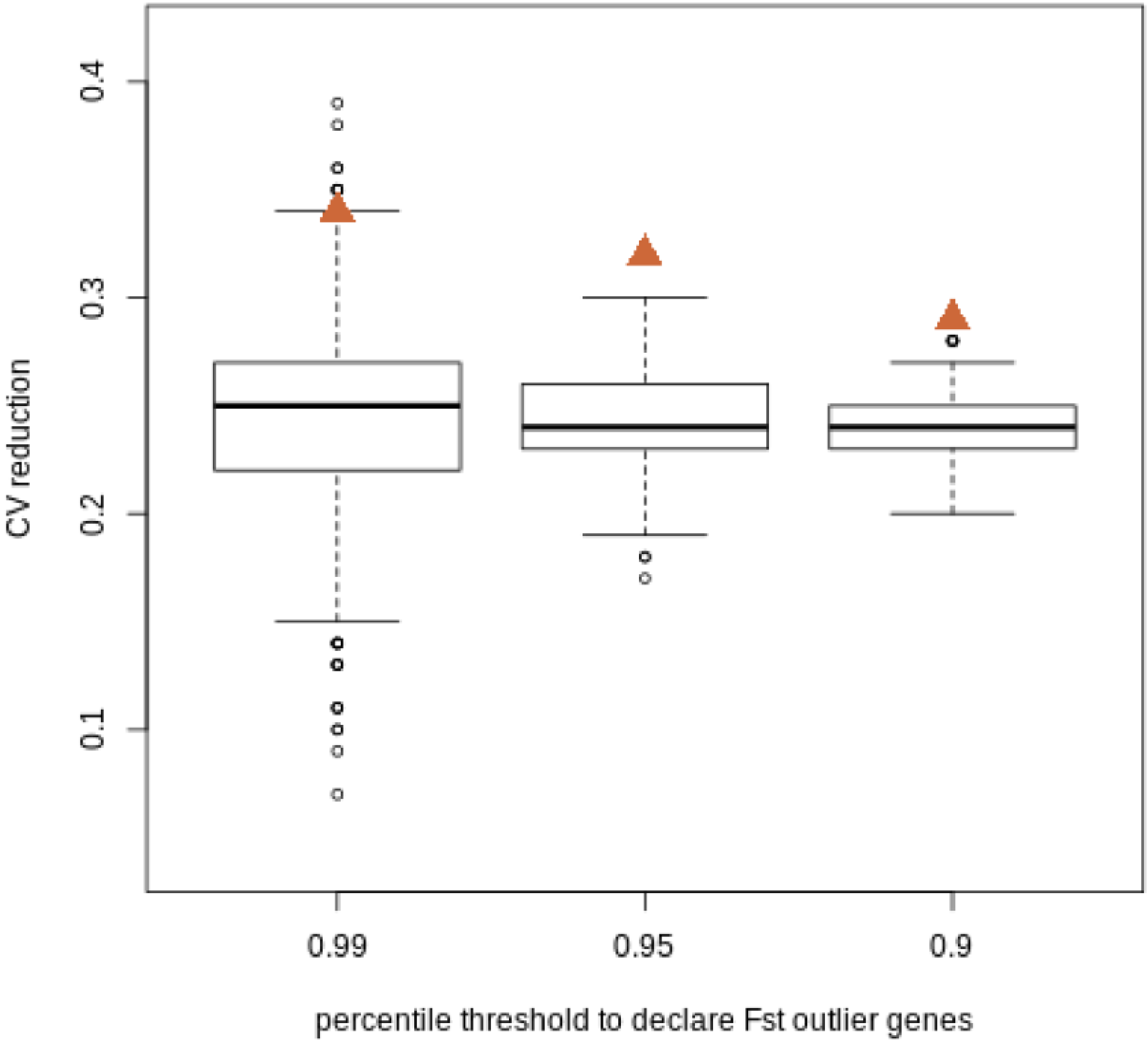

Figure S2. Observed (red triangles) and expected (black boxplots) wild to domesticate reduction of the coefficient of variation in expression (CV). Observed values were calculated on genes with extreme values of domesticate-wild differentiation (FST outliers) at different FST thresholds: 99%, 95% and 90% upper percentiles. Expected values were calculated on 1000 random samples of non outliers genes with sample size equal to the number of outliers at each FST threshold and equal or lower levels of diversity p. Sample sizes: 92, 457 and 913 respectively. CV reduction was calculated as 1-(mean CVCROP / mean CVWILD).
