# Supplementary material for: The Road to Sorghum Domestication: Evidence From Nucleotide Diversity and Gene Expression Patterns
Source: Front Plant Sci. 2021 Aug 30;12:666075. doi: 10.3389/fpls.2021.666075 (PMC8435843; doi:10.3389/fpls.2021.666075)
Supplement: Supplementary file 1 [file Data_Sheet_1.zip › Suplementary_Figure_S3.pdf]

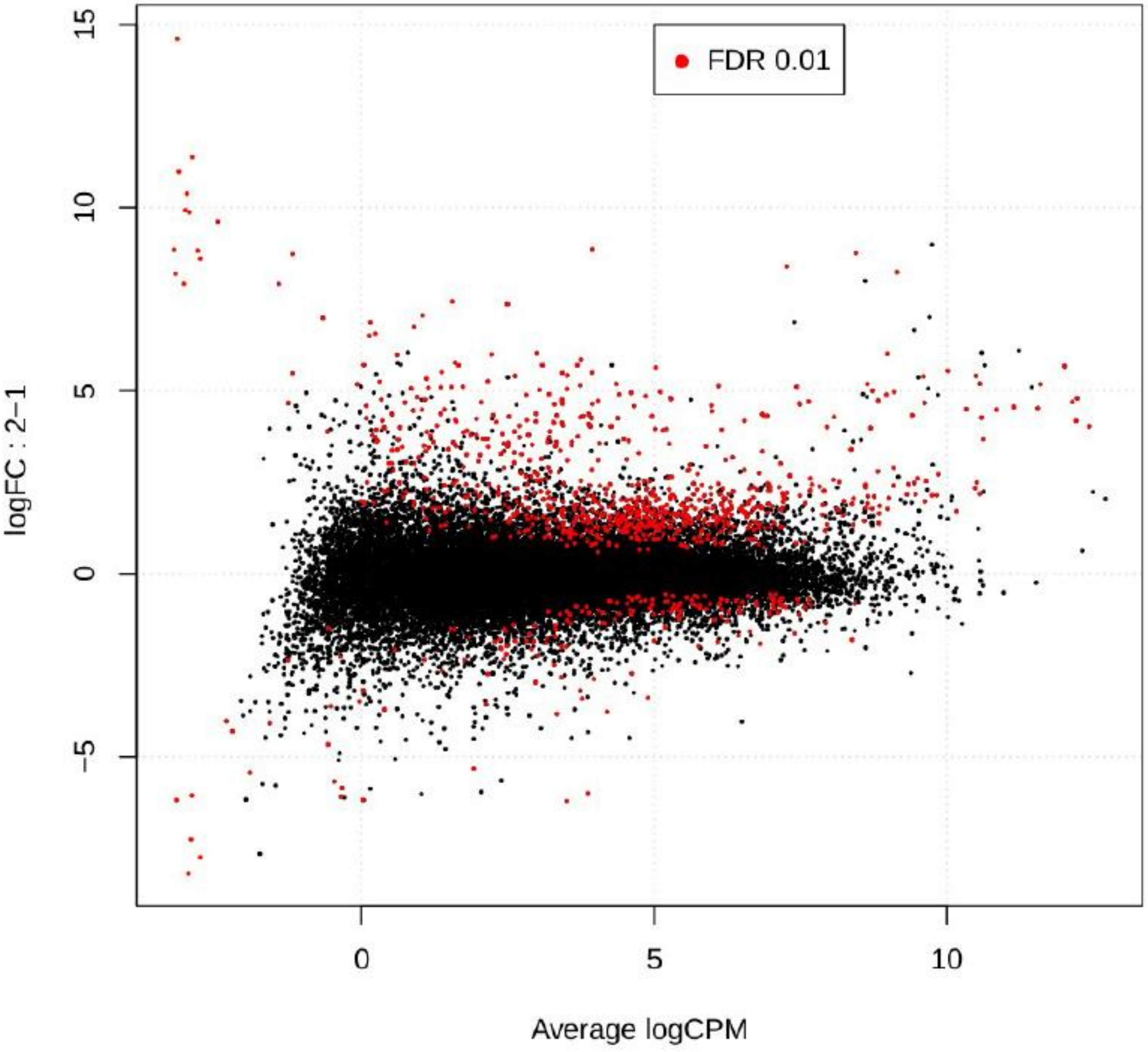

Figure S3. Plot of log-Fold Change (i.e. the log of the ratio of expression levels for each gene between wild and domesticated sorghum) against the log-concentration for count data (i.e. the overall average expression level for each gene across the two groups) for all genes analysed in this study (n= 24646), created with edgeR. Red: genes differentially expressed at 1% FDR (n=949).
