# Supplementary material for: The Road to Sorghum Domestication: Evidence From Nucleotide Diversity and Gene Expression Patterns
Source: Front Plant Sci. 2021 Aug 30;12:666075. doi: 10.3389/fpls.2021.666075 (PMC8435843; doi:10.3389/fpls.2021.666075)
Supplement: Supplementary file 1 [file Data_Sheet_1.zip › Suplementary_Figure_S4.pdf]

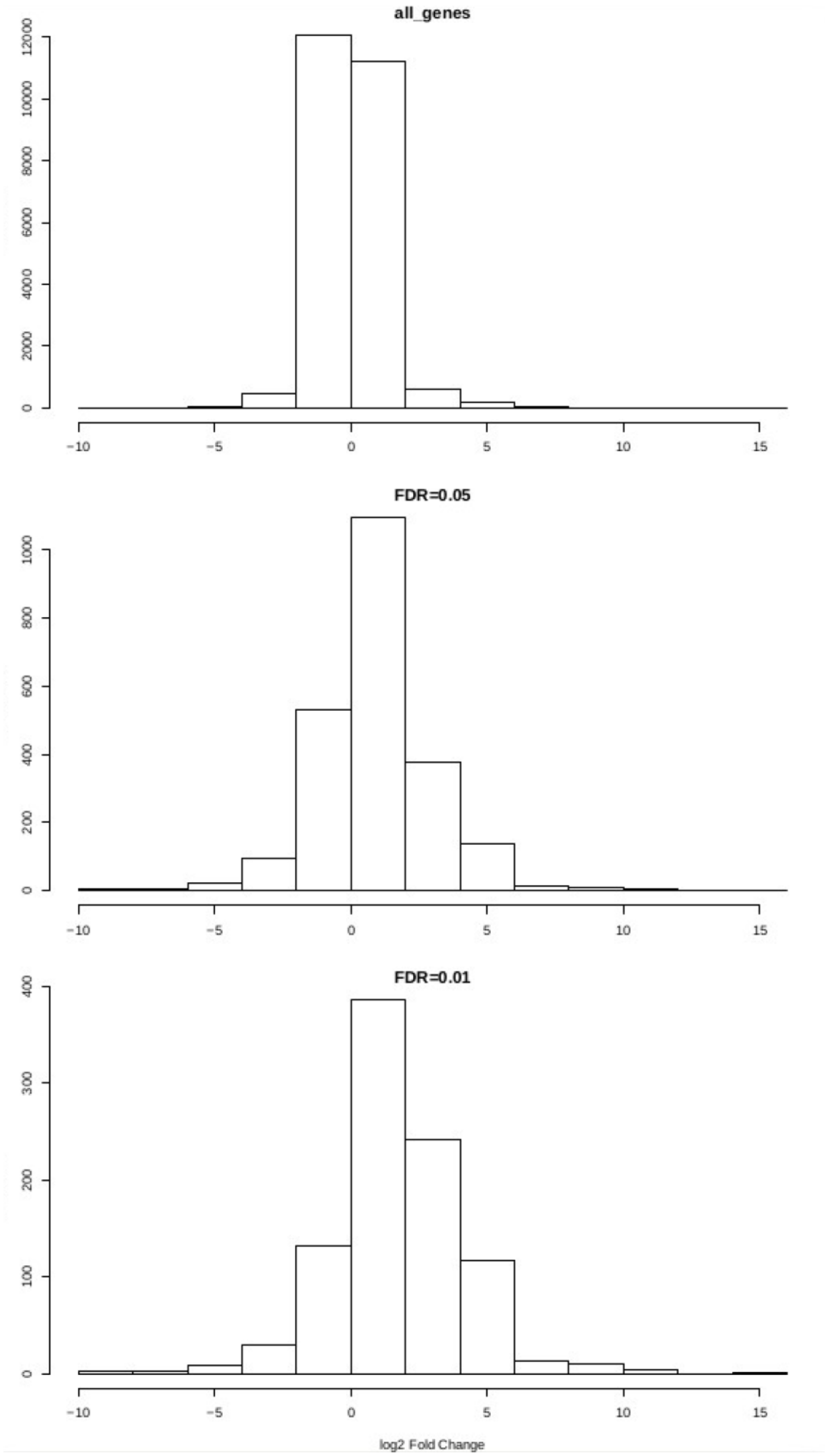

Figure S4. Distribution of fold change (log scale) between wild and domesticated sorghum in different gene sets: all genes (n= 24646, top panel), genes differentially expressed at 5% FDR (n= 2291; middle) and genes differentially expressed at 1% FDR (n=949; bottom).
