# Supplementary material for: The Road to Sorghum Domestication: Evidence From Nucleotide Diversity and Gene Expression Patterns
Source: Front Plant Sci. 2021 Aug 30;12:666075. doi: 10.3389/fpls.2021.666075 (PMC8435843; doi:10.3389/fpls.2021.666075)
Supplement: Supplementary file 1 [file Data_Sheet_1.zip › Suplementary_Figure_S5.pdf]

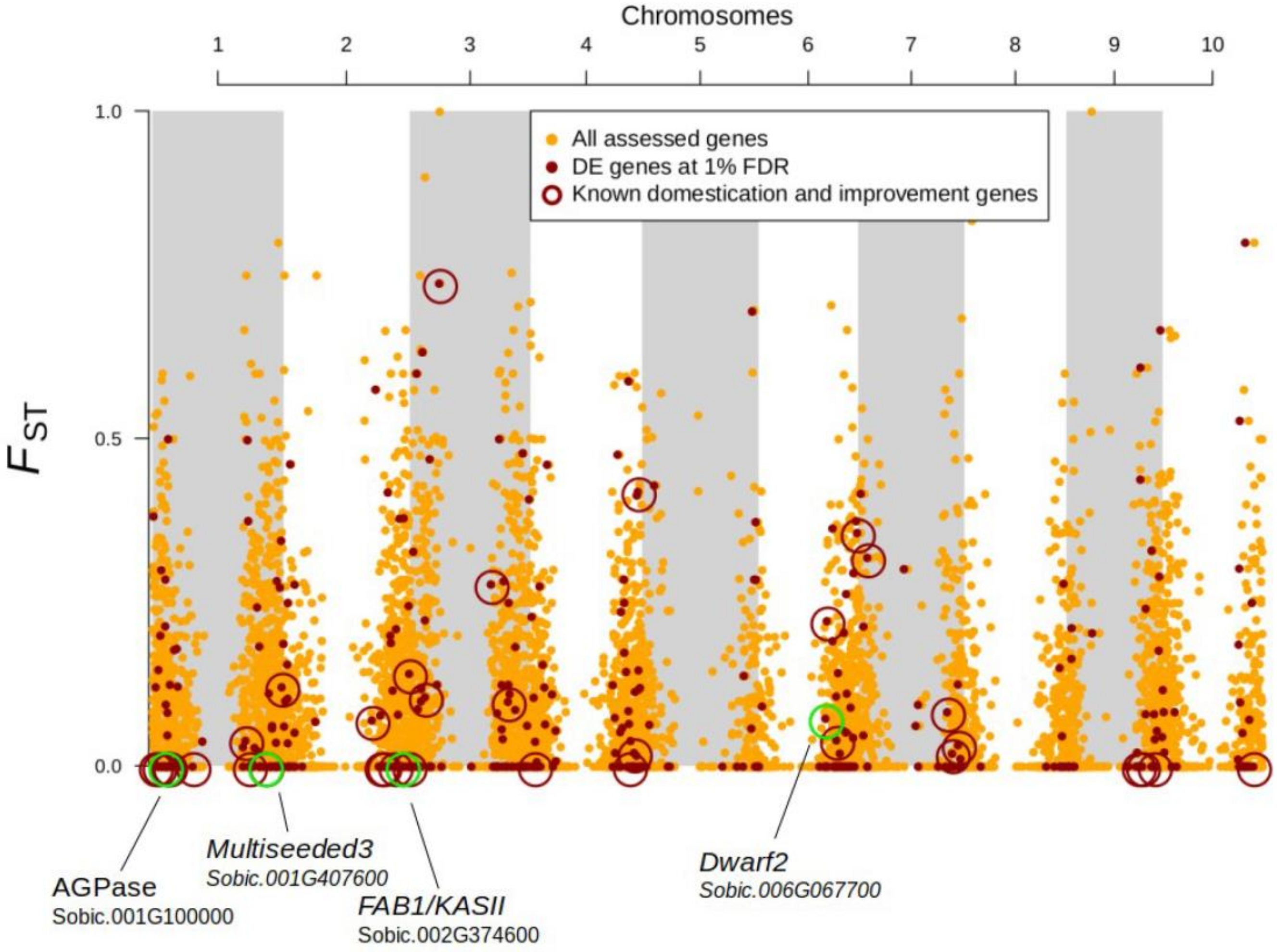

Figure S5. Wild-domesticated FST per gene across chromosomes. Many differentially expressed genes (DE, darkred) show no differentiation between the two pools (FST=0), suggesting that the domestication process has impacted expression but not the nucleotide diversity. Differentially expressed genes that are also known candidate genes for domestication-improvement are listed in Supplementary Table S3 and are the same represented in Figure 2 of the main text.
