# Supplementary material for: The Road to Sorghum Domestication: Evidence From Nucleotide Diversity and Gene Expression Patterns
Source: Front Plant Sci. 2021 Aug 30;12:666075. doi: 10.3389/fpls.2021.666075 (PMC8435843; doi:10.3389/fpls.2021.666075)
Supplement: Supplementary file 1 [file Data_Sheet_1.zip › Suplementary_Figure_S7.pdf]

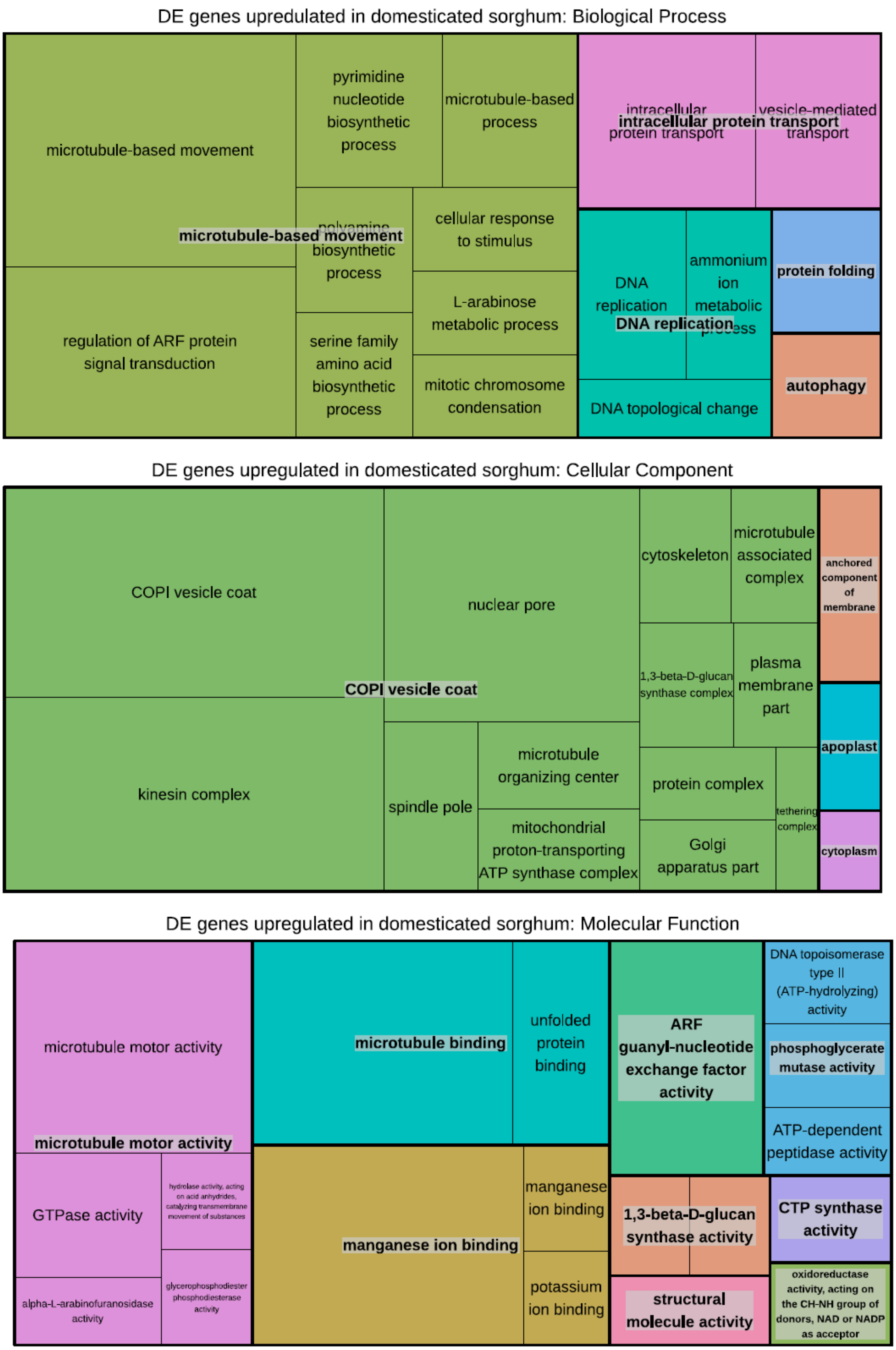

Figure S7. Graphical representation of GO terms associated with genes significantly upregulated in domesticated sorghum at 1% FDR (n=176) made with REVIGO.
