# Supplementary material for: The Road to Sorghum Domestication: Evidence From Nucleotide Diversity and Gene Expression Patterns
Source: Front Plant Sci. 2021 Aug 30;12:666075. doi: 10.3389/fpls.2021.666075 (PMC8435843; doi:10.3389/fpls.2021.666075)
Supplement: Supplementary file 1 [file Data_Sheet_1.zip › Supplementary Text 1.pdf]

Supplementary Text 1. Script to perform the New Tuxedo pipeline, to calculate reads and transcript count from RNA seq data in Sorghum bicolor wild and domesticated accessions

```
#!/bin/bash


##############################################################################
#### Concetta Burgarella - Cirad
####
#### October 2017
####
#### New-tuxedo pipeline: pipeline to prepare gene and transcript count tables to perform a differential
expression
analysis
####
#### Sources: Pertea et al. 2016 Nature Protocols 11:1250, Angélique Berger (Cirad)
####
#### Use:
####
#### Put in the folder where analysis will be done: the scripts (diff_expression_pipeline.sh,
geneIdtogeneName.pl,
prepDE.py) and the sample.list file
####
#### Run the pipeline from the folder the script is with the following command line:
#### qsub -cwd -q normal.q -pe parallel_smp 20 -b y -M concetta.burgarella@cirad.fr -m bea -V -N
DE_pipeline.log ./
diff_expression_pipeline.sh

##############################################################################

# Prepare the sample.list in an automatic way for Arcad samples and BFF samples (for different samples
adapt the
command line or prepare the list manually)
# (run only once)
# cd /gs7k1/projects/arcad_data/sp1_final/sorghum/cleaned_data/
# ls *.gz > /work/burgarella/file.list
# for file in $(cat /work/burgarella/file.list); do begin=$(echo `expr index "$file" E`); echo ${file:(($begin-
1)):4}; done |
tr -d '_' | tr -d '.' | sort > /work/burgarella/samples.list

##############################################################################

# Load necessary modules
module load bioinfo/hisat2/2.0.5 # mapping utility
module load bioinfo/samtools # sort and transform sam to bam
module load bioinfo/stringtie/1.3.3b # assembling and quantifying genes and transcripts

##############################################################################

# Set variables provided by the user
sample_list="samples.list";                                   # list of sample names
echo "File with sample names: " $sample_list;
dos2unix "$samplelist" ;                                      # check format compatibility
input_folder="/gs7k1/projects/arcad_data/sp1_final/sorghum/cleaned_data/";
# localisation (folder) of qstat files
echo "Folder with qstat input files: " $input_folder;
ref_genome="/gs7k1/projects/BFF/sorghum_bicolor/Sorghum_genome_versions/annotation/formated_f
iles/
```

```
Sbicolor_313_v3.1.assembly.fna" ; # fasta file of the reference genome for mapping (why not CDS only???)
ref_annotation="/gs7k1/projects/BFF/sorghum_bicolor/RNAseq/BFF/Sbicolor.gtf" ;
# annotation of the reference genome (it is a gff modified == .gtf prepared by A.B.)

gene_name="ARCAD_BFF" ;
# Name to be given to genes and transcripts not found in the genome annotation.
echo "Name to use for genes and transcripts:" $gene_name;

##########################################################################

# Steps 1, 2, 3 (Pertea et al 2017) [performed in each sample folder (loop on samples)]
# for each sample of the list
for sample in $(cat "$sample_list") ; do
echo "Sample treated now: " $sample ;
echo "Input files: " "$input_folder"*"$sample"[._].* ;
# Create a folder with the sample name
mkdir "$sample" ;
# change directory to the sample folder
cd "$sample" ;
# find and copy compressed files into the sample directory
find "$input_folder" -iregex ".*$sample[._].*" -exec scp {} . \;
# decompress tar.gz files
tar -zxvf *gz ;
# 1. Perform mapping
echo "1. Mapping";
hisat2 -p 20 -x "$ref_genome" -1 *forward* -2 *reverse* -U *single* -S "$sample".sam ; #
SAM includes reads that failed to align
echo "Mapping file created: " *.sam ;
# 2. Sort .sam and transform to .bam (Sort alignments by leftmost coordinates, or by read name when -n is
used)
echo "2. Sort and transform to bam";
samtools sort -@ 8 -o "$sample".sorted.bam "$sample".sam ; # -@ INT = Number of BAM compression
threads to use in addition to main thread [0]
samtools flagstat "$sample".sorted.bam ;
echo "Sorted bam file created: " *.sorted.bam ;
# 3. assemble and quantify genes and transcripts (the output file of this step need a unique, ie per sample,
file
name)
echo "3. Assemble and quantify genes and transcripts";
stringtie -p 10 -G "$ref_annotation" -o "$sample".gtf -l "$sample" "$sample".sorted.bam ;
# -G <ref_ann.gff> Use the reference annotation file (in GTF or GFF3 format) to guide the assembly
process.
# The output will include expressed reference transcripts as well as any novel transcripts that are
assembled.
# [-l <label> name prefix for output transcripts (default: MSTRG)]
echo "Assembled genes and transcripts in gtf file: " *.gtf
cd .. ; # return to the parent directory
done

##########################################################################

# Step 4. Merge individual gtf files (Pertea et al 2017) [to be done in the parent folder]
# create a file with the list of gtf file paths for all the samples wanted to be merged
ls */*.gtf > mergelist.txt ; # gtf files created for current samples
ls /gs7k1/projects/BFF/sorghum_bicolor/RNAseq/BFF/ARCAD/Stringtie/BFF_2013/**/*.gtf >>
mergelist.txt ; #
BFF samples
ls /gs7k1/projects/BFF/sorghum_bicolor/RNAseq/BFF/ARCAD/Stringtie/BFF_2014/**/*L*.gtf >>
mergelist.txt ; #
```

```
BFF samples (some .gtf not wanted)

ls /gs7k1/projects/BFF/sorghum_bicolor/RNAseq/BFF/ARCAD/Stringtie/BFF_2015/**/*L*.gtf >>

mergelist.txt ; #

BFF samples (some .gtf not wanted)
echo "Nb of samples included in the merged list:"
wc -l mergelist.txt # nb of files (should be 782)
# merge (in this step, transcripts not associated to a gene model included in teh reference annotation file
are given a unic
specific name)
# -l <label> name prefix for output transcripts (default: MSTRG)
echo "4. Merge individual gtf files" ;
stringtie --merge -p 20 -G "$ref_annotation" -o "$gene_name"_merged.gtf -l "$gene_name" mergelist.txt ;
echo "Merged gtf file created:" *merged.gtf ;

##########################################################################

# Step 5. Compare transcripts with the reference annotation (Pertea et al 2017) [to be done in the parent
folder]
# adds to each transcripts a class code and the name of the transcript from the reference annotation file
echo "5. Compare transcripts with the reference annotation " ;
/gs7k1/projects/BFF/sorghum_bicolor/RNAseq/BFF/gffcompare/gffcompare -r "$ref_annotation" -o
merged
"$gene_name"_merged.gtf ;
# change the order of designation for 2 columns (what is called "gene_name" becomes "gene_id" and
viceversa)
# this is necessary to get again a format similar to ARCAD_BFF_merged.gtf that merged.annotated had
changed
perl geneIdtogeneName.pl merged.annotated.gtf ; # the output is merged.annotated.gtf.cleanId.gtf
#wc -l merged.annotated.gtf.cleanId.gtf # 972623 (a bit less than BFF_782_merged.gtf...)

##########################################################################

# 6. Estimate transcript abundance and store the results in tables to be read under the R environment [to
be done in the
parent folder]
# needs .bam files, sample.file and merged.annotated.gtf.cleanId.gtf
echo "6. Estimate transcript abundance and store the results in tables" ;
mkdir abundance ;
# estimate abundance on a per sample basis and in a new folder per sample
for sample in $(cat "$sample_list") ; do
echo "Sample treated now: " $sample ;
echo "input files: " "$sample"/"$sample"*sorted.bam ;
mkdir abundance/"$sample" ;
# estimate transcript abundance
# [ -e only estimate the abundance of given reference transcripts (requires -G)]
# [ -B enable output of Ballgown table files which will be created in the same directory as the output GTF
(requires -G, -o recommended)]
# [-l <label> name prefix for output transcripts (default: MSTRG)]
stringtie -e -B -p 10 -l "$sample" -G merged.annotated.gtf.cleanId.gtf -o
abundance/"$sample"/"$sample"_abundance.gtf "$sample"/"$sample"*sorted.bam ;
done
# create tables of gene and transcript abundance with the phyton script provided by stringtie
./prepDE.py -i abundance -g "$gene_name"_gene_count.csv -t "$gene_name"_transcript_count.csv -p E -s
"$gene_name" ;
echo "Count table files generated:" *.csv ;
echo "END OF THE ANALYSIS"
```
